# Enhancing Endoscopic Dacryocystorhinostomy: A Novel Transethmoidal Approach to the Lacrimal Fossa

**DOI:** 10.7759/cureus.92327

**Published:** 2025-09-14

**Authors:** Francesco Giombi, Giovanna Muci, Michele Cerasuolo, Alessandra Di Maria, Luca Malvezzi

**Affiliations:** 1 Otorhinolaryngology, Head and Neck Surgery Unit, Casa di Cura Humanitas San Pio X, Milan, ITA; 2 Otorhinolaryngology, Head and Neck Surgery Unit, Istituto di Ricovero e Cura a Carattere Scientifico (IRCCS) Humanitas Research Hospital, Milan, ITA; 3 Department of Ophthalmology, Istituto di Ricovero e Cura a Carattere Scientifico (IRCCS) Humanitas Research Hospital, Milan, ITA

**Keywords:** chronic dacryocystitis, chronic epiphora, endoscopic dacryocystorhinostomy, endoscopic sinus surgery (fess), lacrimal duct obstruction

## Abstract

Endoscopic dacryocystorhinostomy (EE-DCR) has been established as an effective strategy for managing lower nasolacrimal duct obstructions (L-NLDOs). Although several techniques have been proposed, none have demonstrated superior outcomes. We developed a novel transethmoidal approach to the lacrimal fossa, which embodies the fundamental principles of endoscopic sinus surgery. Images and video are presented to describe the procedure and for educational purposes. Patients with unilateral L-NLDO were evaluated preoperatively (T0), at 6 months (T1), and 12 months (T2) postoperatively. Surgical success was defined as complete (patency on irrigation with symptom resolution) or anatomical (patency with persistent epiphora). Overall, surgical success was achieved in 308/365 (84.4%) patients at T1 and in 317/365 (86.9%) patients at 12 T2. At T1, 155/365 (42.47%) patients achieved complete success, and 153/365 (41.49%) anatomical success. At T2, 215/365 (58.90%) had complete success, and 102/365 (27.94%) had anatomical success. Minor complications (n = 13/365, 5.56%) were managed intraoperatively. The mean procedure duration was 37.40 ± 12.12 minutes. In our experience, this approach guaranteed favorable results and low complication rates, with reasonably short procedural times.

## Introduction

Endoscopic endonasal dacryocystorhinostomy (EE-DCR) has become the gold standard for the management of nasolacrimal duct obstruction. The technique consists of surgically bypassing the blockage in the lacrimal drainage system by the creation of a stoma between the lacrimal sac (LS) and the lateral nasal wall. Several approaches to EE-DCR have been proposed, often requiring extensive mucosal flaps to adequately expose the lacrimal bone (LB) [1,2]. Nevertheless, evidence regarding which technique is the most effective remains lacking [3]. This technical report aims to present a novel and feasible approach to EE-DCR, based on the identification and dissection of key endoscopic landmarks, thereby pursuing a transethmoidal pathway to simplify the exposure of the lacrimal fossa.

## Technical report

Overview

**Figure 1:**
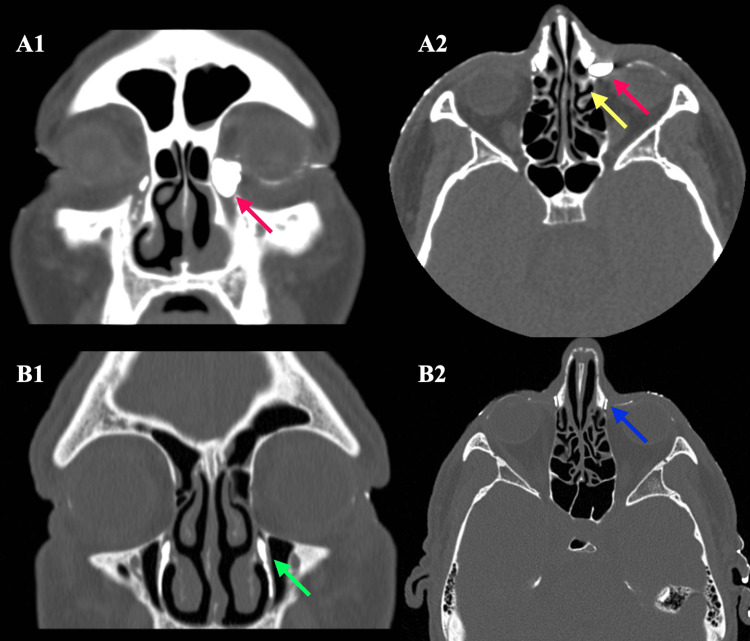
CT scan with iodine contrast enhancement of the lacrimal pathway. (A) Lower nasolacrimal duct obstruction (A1: coronal view; A2: axial view) resulting in a mucocele of the left lacrimal sac. (B) Contrast medium correctly draining onto the inferior lacrimal pathways (Hasner’s valve). As no signs of lower nasolacrimal duct obstruction are visible, different causes of epiphora should be investigated (B1: coronal view; B2: axial view). Red arrow: mucocele of the lacrimal sac; yellow arrow: insertion of the uncinate process; green arrow: inferior lacrimal drainage pathway; blue arrow: common canaliculi.

This is a single-center, retrospective, observational study. Consecutive patients who underwent EE-DCR for unilateral primary acquired nasolacrimal duct obstruction were enrolled between January 2020 and 2024. Exclusion criteria included upper obstruction (punctum or canaliculi), previous DCR and/or nasal surgeries, diagnosis of secondary obstruction, or being affected by other ocular diseases. An ophthalmologic evaluation and a preoperative CT scan were required for inclusion (Figure 1).

Included patients were required to undergo preoperative (T0) and follow-up assessments (T1: 6 months; T2: 12 months). At each examination, they underwent lacrimal irrigation and were asked to rate the frequency of tearing by filling in the Munk score (range: 0-4) (Table 1) [4], as well as the number of episodes of dacryocystitis over the last six months. Dacryocystitis was defined as the presence of purulent discharge or swelling at the medial canthus, with or without fever, requiring topical treatment or systemic antibiotic therapy [5].

**Table 1:** Munk scale for grading epiphora.

Grade	Munk scale
0	No epiphora
1	Epiphora requiring dabbing fewer than twice a day
2	Epiphora requiring dabbing 2–4 times a day
3	Epiphora requiring dabbing 5–10 times a day
4	Epiphora requiring dabbing >10 times a day or constant epiphora

Complete success was defined as the resolution of symptoms (Munk scale = 0) and patency on irrigation. Anatomical success was defined by patency on irrigation associated with persistence of mild-to-moderate epiphora (Munk scale = 1-3) and absence of dacryocystitis. Surgical failure was defined as blockage upon irrigation, possibly associated with persistent severe epiphora (Munk scale = 4) or recurrence of dacryocystitis.

Surgeries and outpatient evaluations were performed by the same equipe. Intraoperative complications were extrapolated from surgical charts. The duration of the procedures was expressed by mean, standard deviation, and range for the single-operated side.

Statistical analysis was performed using SPSS Statistics for Macintosh, Version 28.0 (IBM Corp., Armonk, NY, USA). Categorial variables were expressed by numbers and percentages and compared through the chi-square test. Paired parametric data were compared through a paired-samples t-test.

Surgical technique

**Figure 2:**
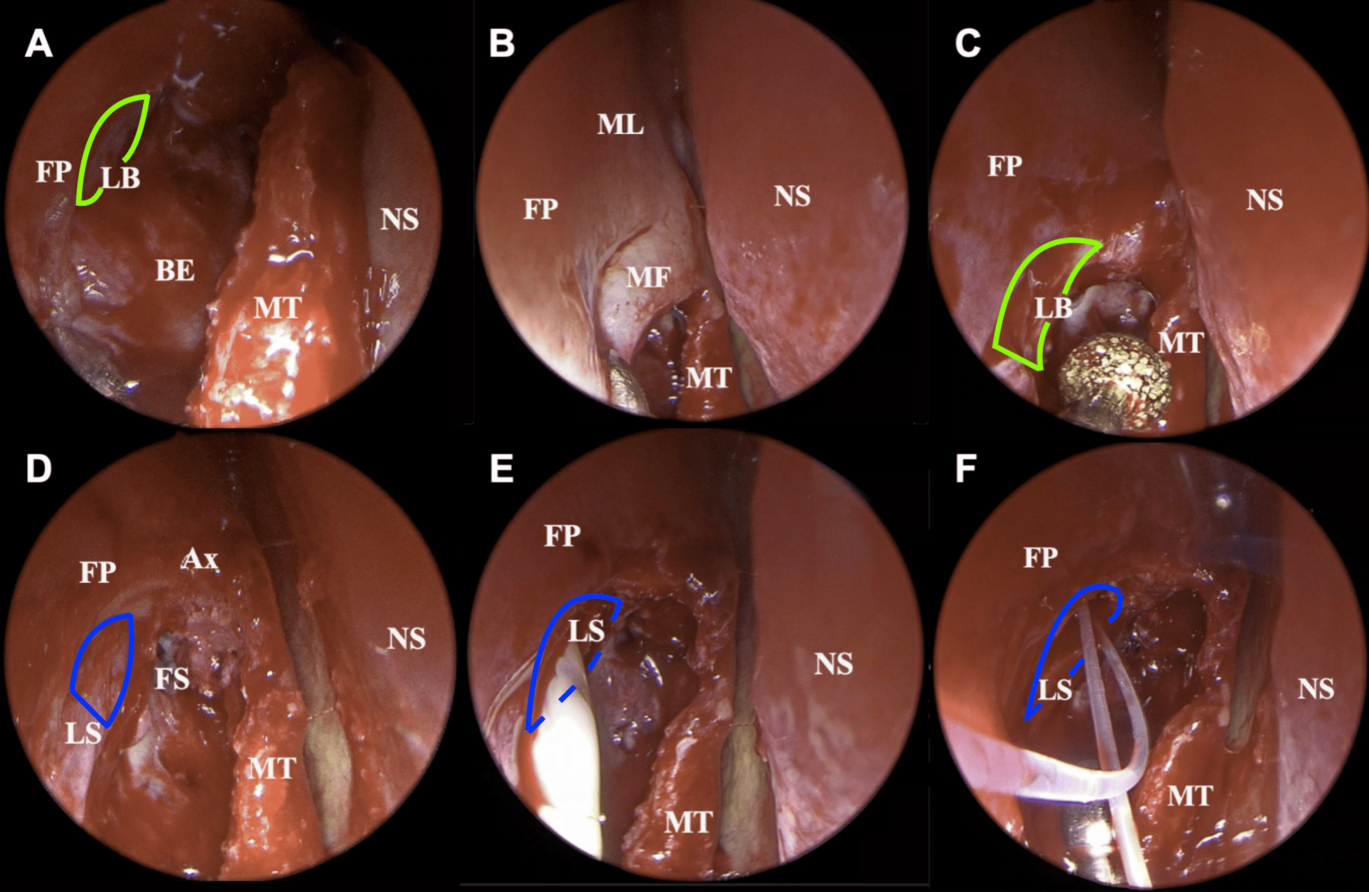
Surgical pearls. (A) Exposure of the medial aspect of the lacrimal bone after removal of the vertical portion of the uncinate process and agger nasi opening. (B) A thin “C-shaped” mucosal flap of approximately 5 mm is removed from the frontal process of the maxillary bone to guarantee wider exposure. (C) Drilling of the lacrimal bone performed with a diamond burr. (D) Adequate exposure of the lacrimal fossa is achieved with minimal bone drilling. (E) Mucopurulent discharge after lacrimal sac incision. (F) Bicanalicular silicone stent intubation. Areas of interest have been highlighted. Green line: lacrimal bone; blue line: lacrimal sac. BE: bulla ethmoidalis; FP: frontal process of the maxillary bone; LB: lacrimal bone; NS: nasal septum; MT: middle turbinate; MF: mucosal flap; ML: maxillary line; FS: frontal sinus; LS: lacrimal sac; Ax: axilla of the middle turbinate

A retrograde inferior uncinectomy with a backbite forceps was performed. Removal of the vertical portion of the uncinate process (UP) provided access to the natural ostium of the maxillary sinus, whose anterior margin is defined by the nasolacrimal duct. The LB was identified after removing the cranial portion of the UP, as these two anatomical structures insert closely into the lateral nasal wall (Figure 2A). Using a Cottle knife, a thin “C-shaped” mucosal flap approximately 5 mm thick was removed from the frontal process of the maxillary bone (Figure 2B). Using a diamond burr and a 45° nasal endoscope, the LB was drilled with a gentle superior-inferior motion, exposing the lacrimal fossa cranially to caudally. Simultaneously, the posterior margin of the frontal process of the maxillary bone was removed using the posterior edge of the drill in a gentle posterior-anterior motion (Figure 2C). These maneuvers ensured a proper exposure of the LS, which was confirmed by palpation with a Bowman probe (Figure 2D). The LS was then incised with a Beaver blade to create a wide nasolacrimal stoma. Possible lacrimal mucoceles were also drained during this step (Figure 2E). It is paramount that the stoma is as wide as possible to ensure the correct exposure of the common lacrimal punctum from the nasal cavity. Finally, a bicanalicular stent was positioned and maintained in place until local healing was achieved, at least three weeks postoperatively (Figure 2F).

A surgical video showing technical pearls has been attached for educational purposes (Video 1).

**Video 1:** Surgical technique. This narrative surgical video clip highlights the key principles of our approach, emphasizing the most critical steps for educational purposes.

Surgical outcomes

Overall, 365 patients (76.43% female, n = 279/365; mean age = 45.92 ± 32.11 years) were included. Preoperatively, the mean Munk score and the dacryocystitis rate were 3.63 ± 1.65 and 72.33% (n = 264/365), respectively. Subjective outcomes were significantly improved at both timepoints; conversely, no difference was observed at 12 months compared to the 6-month examination (Tables 2, 3).

**Table 2:** Patient-reported outcomes at each timepoint. The mean Munk score and the prevalence of dacryocystitis episodes in the overall population are reported. SD: standard deviation

Timepoint	Munk score ± SD	Dacryocystitis rate
T0	3.63 ± 1.65	72.33% (264/365)
T1	1.94 ± 1.29	14.79% (54/365)
T2	1.59 ± 1.21	12.33% (45/365)

**Table 3:** Pairwise analysis between patient-reported outcomes at each timepoint. ^a^ = Paired samples t-test. Difference of the means ± standard deviation is displayed; ^b^ = Chi-square test. Results are reported in terms of chi-square statistics. P-values are displayed between brackets. Bonferroni correction for multiple comparison was applied.

Timepoint	Munk score (p-value)^a^	Dacryocystitis rate (p-value)^b^
T0 vs. T1	1.69 ± 1.53 (<0.001)	245.72 (<0.001)
T0 vs. T2	2.04 ± 1.75 (<0.001)	269.14 (<0.001)
T1 vs. T2	0.35 ± 1.21 (0.891)	0.95 (0.331)

Surgical success was observed in 308 (84.38%) patients at T1, including 155 (42.47%) patients with complete success and 153 (41.49%) patients with anatomical success. At T2, 317 (86.85%) patients achieved surgical success, with 215 (58.90%) showing complete success and 102 (27.94%) showing anatomical success. In all cases, surgical failure subtended the obliteration of the lacrimal stoma. Between patients who experienced failure (T1: 15.62%, n = 57/365; T2: 13.15%, n = 48/365), most cases were due to scarring adhesions (T1: 63.16%, n = 36/57; T2: 85.42%, n = 41/48), whereas others to granulations (T1: 36.84%, n = 21/57; T2: 14.58%, n = 7/48) (Table 4).

**Table 4:** Surgical outcomes at postoperative timepoints.

Timepoint	Success		Failure
	Complete	Anatomical	
T1	155/365 (42.47%)	153/365 (41.49%)	57/365 (15.62%)
T2	215/365 (58.90%)	102/365 (27.94%)	48/365 (13.15%)

Intraoperative minor complications were observed in 13 (3.56%) patients, including 10 (2.74%) who experienced breach of the lamina papyracea with orbital fat exposure and three (0.82%) who experienced intraoperative bleeding. In all cases, complications were successfully managed intraoperatively. The mean duration of the procedure was 37.40 ± 12.12 minutes (median = 37 minutes; range = 15-71 minutes).

## Discussion

Endoscopic dacryocystorhinostomy was pioneered by Wormald et al., who proposed an anterior approach to the lacrimal fossa through the anterior frontal process of the maxillary bone (e.g., “powered DCR”) [1]. In our experience, his technique presented some crucial limitations. The creation of a wide mucosal flap is a labor-intensive process, extending surgical duration and delaying the healing of the lateral nasal wall. As the access is achieved anteriorly through the frontal process of the maxilla, a substantial amount of bone must be drilled to adequately expose the LS, which can induce heat damage to surrounding tissues and potentially contribute to abnormal healing. In recent years, vertical uncinectomy has been implemented in EE-DCR, although most described techniques still follow an anterior path to the lacrimal bone, thus often requiring wide maxillary osteotomies [6,7].

In this scenario, we presented a novel, feasible approach to the lacrimal fossa. The key aspects of our technique include: (i) complete vertical uncinectomy and removal of the agger nasi, hence pursuing a minimally invasive path to expose the lacrimal bone; (ii) conservative (~5 mm) mucosal flap removal; (iii) limited drilling of the frontal process of the maxilla and lacrimal bone inferior to the axilla of the middle turbinate; (iv) wide dacryocystorhinostomy; and (v) canalicular intubation. These steps differed from the traditional technique as the lacrimal fossa was exposed with minimal drilling, without removing significant parts of the frontal process of the maxilla, and without leaving bony walls exposed. Postoperative outcomes seemed promising: overall, we observed a significant improvement in patient-reported outcomes (e.g., epiphora, dacryocystitis) within six months (Table 3). At T1, 308 (84.38%) patients achieved surgical success, with 155 (42.47%) complete and 153 (41.49%) anatomical success. By T2, 317 (86.85%) procedures were successful, including 215 (58.90%) complete and 102 (27.94%) anatomical (Table 4). These results align with reported surgical success rates of 80% to 95% [8-11]. Moreover, the average duration of the procedure was reasonably low.

In the overall sample, we observed 13 (3.56%) intraoperative complications, mainly due to lamina papyracea breach, all managed intraoperatively, resulting in periorbital hematoma. Only three (0.82%) patients experienced intraoperative bleeding due to anterior ethmoidal artery dehiscence. Although the management of these cases may be challenging for inexperienced surgeons, they were all effectively treated endoscopically, and in neither case was a medial canthotomy required to stop the bleeding. Generally, EE-DCR may be considered safe, although minor complications have been reported, ranging from 3.2% to 13.7% [12-14]. Intraoperative complications include local bleeding, orbital wall injury, injury to canaliculi due to inappropriate probing, and, exceptionally, cerebrospinal fluid leak [15,16]. Our minimally invasive approach may reduce intraoperative complications while maintaining comparable postoperative outcomes.

Limitations of this approach include its retrospective design; therefore, further analyses with larger cohorts and longer follow-up periods are needed to confirm the reproducibility of the results. In parallel, conducting multivariate analyses is needed to exclude possible confounding factors. Furthermore, the inclusion of a population derived from a single institution could have induced a selection bias, as anatomical variations in the height of the lacrimal fossa can occur across different ethnicities [17]. From our experience, to reduce the risk of complicated LB exposure, a preoperative CT scan is recommended to accurately locate the LB’s projection on the lateral nasal wall. From a technical point of view, the main limitation of this approach is the preference for experienced endoscopic surgeons. This is because it integrates the concepts of lacrimal surgery with those of functional nasal surgery to expand the operating field. As a result, this technique is more suitable in cases when the patient, besides DCR, also requires comprehensive procedures (e.g., full-house functional endoscopic sinus surgery) due to sinonasal comorbidities. Accordingly, we believe that transethmoidal EE-DCR should be practiced and taught in referral academic environments. Nevertheless, future research comparing operative outcomes between experienced surgeons and trainees, as well as analyzing learning curves, will be needed to address these issues.

## Conclusions

Traditional techniques for EE-DCR rely on wide osteotomies, which provide labor-intensive and cumbersome access to the lacrimal fossa. In this technical report, we describe a novel and feasible technique that simplifies surgical access via a minimally invasive approach. As it incorporates the principles of endoscopic sinus surgery, this procedure is particularly suitable for cases requiring functional sinonasal surgery to address sinonasal comorbidities. Safety was confirmed, as no major intraoperative complications were observed, and the limited number of minor adverse events was feasibly manageable intraoperatively. Future research should include randomized trials to compare different approaches in multicentric settings, thereby confirming the reliability of our results and supporting broader implementation in standard clinical practice.
